# Testing urine for drugs

**DOI:** 10.1155/S1463924692000191

**Published:** 1992

**Authors:** D. Catlin, D. Cowan, M. Donike, D. Fraisse, H. Oftebro, S. Rendic

**Affiliations:** 1 Departments of Medicine and Pharmacology University of California Los Angeles USA; 2 UCLA Medical Center 650 Circle Drive South Los Angeles California 90024 USA


Journal of Automatic Chemistry, Vol. 14, No. 3 (May–June 1992), pp. 85–92

*International Federation of Clinical Chemistry (IFCC)*

### Testing urine for drugs

**D. Catlin*, D. Cowan, M. Donike, D. Fraisse, H. Oftebro and S. Rendic**

** Corespondence to Dr Don H. Catlin, Departments of Medicine and Pharmacology, University of California, Los Angeles, UCLA Medical Center, 650 Circle Drive South, Los Angeles, California 90024, USA*

### Introduction

The rising demand for drug testing comes from pressure from society to stem the spread of substance abuse and to provide people with greater protection. In response to this demand, urinalysis testing programmes have been implemented by a variety of organizations, such as business and industrial employers, the transportation industry, police and fire departments, the military, and sports [1–7]. The drugs most commonly tested for are marijuana, cocaine, amphetamines and narcotic analgesics. Some programmes also test for benzodiazepines, barbiturates and phencyclidine.

A common denominator is the widely accepted notion that drug testing will identify the primary offenders. The extent to which this objective is achieved is most appropriately addressed by specialists in behavioural medicine and sociology. The scientific basis for urinalysis programmes is in the domain of clinical and analytical chemistry.

The analysis of body fluids for drugs takes place in two distinctly different environments: the medical model and the penalty model. In the former, and in the context of patient care, the physician requests the test and the patient is both fully co-operative with the sample collectors and highly motivated to provide the ideal sample. In the latter case, the request usually comes from a non-medical authority and it may not be in the best interest of the individual to submit a valid sample. In the medical model the information obtained from the test is used to assist the physician in the care and management of the patient. In contrast, in the penalty model the results are used to impose some penalty to the individual. This fundamental difference, together with legal imperatives, has broad implications for the overall design and implementation of a drug testing programme.

Although it may seem counter-intuitive, testing in the penalty model is far more demanding and difficult than with the medical model. For example, if a physician sends a sample from an unconscious patient to the laboratory for a rapid screen, and, one hour later, the report is positive for opiates by immunoassay the physician will use this information to tailor treatment to an opiate overdose, as opposed to, say, a barbiturate overdose. It is relatively unimportant to know if the primary drug is morphine or codeine because the clinical implications are very similar. In contrast, if the urine came from a pre-employment screening programme, it would be of utmost importance to know if the positive test was due to the consumption of heroin, a dangerous and illicit drug, or codeine, a drug which is widely available and commonly prescribed for pain. In fact, since urinary morphine could arise from the ingestion of morphine, heroin, or codeine the complete analysis of this sample requires a specific and quatitative analysis for all metabolites of all three substances. Furthermore, this type of analysis virtually requires gas chromatography-mass spectrometry (GC-MS), a technique which is inherently difficult, labour intensive, and expensive. Thus the complete analysis of a complex sample is generally beyond the traditional scope of hospital- or clinic-based clinical chemistry laboratories, and serves to emphasize that the clinical chemist who wishes to extend his practice to drug testing must be prepared for a substantial increase in analytical instrumentation and technical training. Further amplification of this point appears in numerous recent publications and reviews [3,4,8].

Additional factors which must be considered in the testing with penalties model include constant scrutiny by the legal community, the activity of regulatory agencies, scrupulous attention to chain of custody both within and without the laboratory, space allocation to provide for specialized functions and security, and development of substantial knowledge regarding the pharmacology, pharmacokinetics and metabolism of the substances. In order to accommodate these considerations the traditional laboratory must reorganize administrative procedures, job descriptions, security, laboratory protocols and space allocation.

Urine testing of sportpersons (doping control) differs from that of employees in that the main reason to test is to maintain fair play by prohibiting the use of performance-enhancing drugs, as opposed to protecting safety or productivity on the job by prohibiting street drugs. In marked contrast to testing for the so-called 'drugs of abuse', the menu of drugs tested for by sport authorities is both comprehensive and includes several classes of substances [1,9]. Some banned substances are also produced endogenously (for example testosterone), and in the case of several substances the ban is based on quantification. Detection of the administration of exogenous testosterone requires special testing techniques [8,10,11].

The current list of substances banned by the International Olympic Committee consists of six groups [8], i.e. the stimulants where several classes are found such as the psychomotor stimulants (for example amphetamine), the sympathomimetics (for example ephedrine), and the analeptics (for example strychnine, caffeine). The other groups are narcotics, anabolic steroids, beta-blockers,

© IFCC 1992

diuretics, and certain peptide hormones. This classification is based on the expected effect of the drugs on the athlete, as opposed to their chemical structure and properties.

### Regulatory considerations

Legal review can be expected [12], so laboratory directors should be familiar with the relevant local, national and international laws. In general, such reviews are designed to find fault with any aspect of the system, and, in particular, the authority to test, provisions for individual consent to undergo testing, collection procedures, chain of custody, sample chemistry, instrument maintenance, tuning, and calibration records, quality control, the analytical data consisting of chromatograms and spectra, sample storage, qualification of laboratory personnel, confidentiality, and appeal process. Laboratories are advised to review carefully the documents which describe the authority to test and the details of the client's protocol. The best defence for an unfavourable legal decision is excellence in analytical chemistry, together with a complete set of written procedures covering all aspects of the testing programme, and documentation showing that all procedures were followed.

Various countries and scientific organizations either have produced or are in the process of preparing guidelines to regulate drug testing laboratories [2,4–6]. These are designed to protect the person tested ('testee') by improving the quality of the work product by emphasis on quality assurance, quality control, proficiency testing, and documentation. In some countries the guidelines are incorporated into accreditation or certification programmes [5]. Consumers of laboratory services are encouraged to use only those that are certified or accredited.

### Sample collection procedures

Prior to urine collection the testee is offered the opportunity to declare all medications and related substances taken recently. This information becomes part of the documentation, and, in the event of a positive test and legal action, it is used by the certifying scientist to evaluate the possibility of analytical interferences or inadvertent use of a banned substance.

It is essential to ensure that the urine tested is authentic urine from the designated individual. Experience has shown that some individuals will attempt to evade submitting authentic urine by techniques such as concealing bladders and tubing under their clothing and even introducing 'clean' urine into their bladder just before the test [13]. To help exclude these measures, and other forms of substitution, the testee should remain under the constant supervision of at least one member of the drug testing team from the time of notification to the time of urination. Next the drug testing official must verify the identity of the testee by requesting a passport, driver's licence, or otherwise accepted document with photograph. Finally, the actual urination should be directly observed by a drug testing team member of the same gender [15]. Some organizations go to further extremes and require removal of all clothing. Others do not directly observe urination, but use temperature-sensitive containers [5].

To ensure sample integrity, the urine collection containers should be individually sealed. The testee is given the opportunity to select any container which is used to collect or ship the specimen. No one but the testee should handle the urine in the container and/or the bottles until they are sealed. The testee and officials all sign a declaration that protocol was followed to their satisfaction. The laboratory should not know the identity of the testee, therefore urine samples are forwarded to the laboratory identified only by a number. The organization retains the confidential master code which links each number to an individual's name. To exclude all varieties of sabotage, the collection area should be secure with restricted access.

Because there are manipulations which affect renal excretion and the urinary concentrations of the forbidden drugs, some drug testing programmes require that the pH and specific gravity be within a specified range and may require holding the testee to provide additional urine that fulfills the criteria. For example the extretion of many nitrogen-containing drugs depends on urine pH. If the urine is alkaline, certain stimulants are excreted less, therefore a testing program may not accept a sample if the pH is 7·5 or greater. Another example is that of dilution by diuretics or excessive liquid intake, such that 1·005 may be the lowest acceptable specific gravity. These parameters may be measured with a dipstick on the urine remaining in the container after all bottles have been sealed, thus excluding the possibility of contamination by the dipstick. In some drug testing programmes the specific gravity is not measured at the collection site. Some laboratories measure specific gravity and/or creatinine before proceeding with the analysis, and, they may use this data to determine if the sample is valid. The laboratory must receive a sufficient volume to complete all tests. Most protocols define a minimum acceptable volume.

For the additional protection of the testee, many organizations require the division of the urine sample into two parts, A and B, to be sealed individually. The laboratory ordinarily receives both, saves the B intact, analyses the A, and reports the results to the organization.

### Chain of custody

To ensure that the urine tested suffered no contamination, tampering, or mislabelling, the chain of custody begins at the collection site and ends with the final report. The sample is handled at first only by the testee until sealed, then by collection site officials, transportation personnel, and laboratory technicians. The control system must guarantee integrity of the specimens from the moment of submission of the urine until the conclusion of the analysis. Each transfer must be documented, including within-laboratory transfers.

Experienced couriers are recommended for the transportation of samples to the laboratory. A chain of custody form and manifest are initiated at the collection site. The laboratory checks the custody form, examines the package for evidence of tampering, and then accepts custody of the package. After opening the package the samples are inspected individually and checked against the manifest. At this juncture the laboratory initiates its internal chain of custody. Once the chain is initiated, only qualified laboratory personnel may work with the sample and no unauthorized visitors are allowed in the work area. The laboratory must be able to give exact documentation on such details as where a certain sample was located at a given time and the identity of the person handling the sample at the time in question. The samples should be stored at a maximum of 4 °C in a locked area.

### Analytical approach

Urinalysis for banned drugs must be done using methods that give firm evidence because of the consequences to lives, careers, and reputations which follow reports of positive analytical findings. A screening test divides the samples into two categories: a large group of analytically negative samples, and a smaller group that requires further analysis (confirmation test). In the latter case, the screening data indicate that a banned substance or its metabolites may be present and provide tentative identification. The principles of decision theory have been used to calculate the predictive value of screening and confirmation tests [14]. Confirmatory tests are time-consuming, complex, and have one main goal: they provide data for the final, unequivocal identification of the banned substance or metabolites. In addition, confirmatory tests are used to confirm the identity of the biological sample (for example that the urine used for the second analysis is the same as the one used for the first analysis), to exclude clerical errors, and to confirm that the analysis is reproducible.

### *Sample preparation*

Glassware must be scrupulously clean. False positive results have been traced to soap residues [4]. To avoid contamination with phthalates nothing but glass or Teflon should come into contact with the sample. The purity of the solvents and reagents should be appropriate to the analysis. Sample preparation is specifically designed in an attempt to optimize the detection of each chemical class of substance. If the drugs and metabolites are excreted as conjugates (sulfates or glucuronides), a hydrolysis step is necessary prior to extraction. Compared to acid hydrolysis, enzymatic hydrolysis is usually less destructive to deconjugated products. Polar compounds containing hydroxyl, keto, acid, or amine functions are usually converted to less polar and more volatile derivatives. The most common derivatizations are trimethylsilylation, trifluoroacylation, and methylation. Many unconjugated nitrogen-containing compounds may be extracted with diethyl ether at pH greater than 12, separated by GC, and detected with a nitrogen phosphorus selective detector (NPD). Acidic compounds, for example barbiturates and benzodiazepines, require extraction at appropriate acidic pH. Amphoteric compounds, such as morphine, are extracted most efficiently at their isoelectric point with polar solvents such as ether and propan-2-ol. Conjugated nitrogen compounds (beta-blockers, opiates, hydroxylated phenylalkylamines) may be extracted after hydrolysis with diethyl ether, derivatized by trimethylsilylation and/or trifluoroacylation, separated by GC, and detected by NPD. Many diuretics are acidic compounds and are extracted at a pH lower than 2. Because of their low volatility and thermolability they are not amenable to GC analysis without derivatization. Diuretics may be screened for by HPLC with UV detection or by GC and/or by GC-MS.

### *Screening tests*

Most of current screening tests are based on immunoassay or chromatographic techniques.

*Immonoassay (IA)*: This is the most commonly employed screening test [3]. A variety of IA-based methods have been developed and automated. One common feature of IA is the utilization of antibodies with specificity for the drug and/or metabolite and closely related substances. Since the antibodies cross-react with substances which are similar in structure to the target substance, the analytical results are neither unambiguous nor strictly quantitative and must be confirmed. A variety of interferences have been described (e.g. [15]).

*Chromatography*: The different kinds of chromatography (for example TLC, GC, LC) are basically separation techniques used to resolve complex biological mixtures. With appropriate physico-chemical detection and with strict standardization of experimental parameters identification and quantitation can be achieved.

(1) Thin layer chromatography (TLC) is a common screening test for drugs of abuse. After an extraction at controlled pH, a tentative identification of drugs can be made based on physico-chemical characteristics (Rf values) and colour reactions.

(2) High performance liquid chromatography (HPLC) is ideally suited for thermolabile and polar substances. It is most commonly used in a modification known as reverse phase chromatography. The conditions may be adjusted to solve a wide range of analytical separation problems. The available choice of detection—UV/visible, fluorescence, electro-chemical, chemical detection, and MS—will allow sensitive group or even substance detection.

(3) Gas chromatography (GC) will separate thermostable drugs that are sufficiently volatile to be eluted from the analytical column. Fused silica capillary columns provide the necessary high resolution to separate extremely complex mixtures or biological matrices. Polar substances need derivatization prior to GC. With element-specific detectors (for example NPD) or in combination with a MS, selective and sensitive detection of a wide variety of drugs is possible.

### *Confirmation tests*

In confirmation tests the unknown and the standard undergo simultaneous sample preparation from extraction to derivatization followed by analysis. Evidence of the presence of a compound includes multiple measures of similarity, such as instrumental analysis data and relative amounts (or concentrations) of multiple urinary metabolites.

Although several approaches may be used to confirm the presence of substances in urine, gas chromatography-mass spectrometry (GC-MS) is currently the method of choice for unambiguous identification. It is the combination of two techniques, where the MS is used as a detector for the GC. GC separates the components of a mixture and introduces them one by one into the mass spectrometer, which records a mass spectrum or 'fingerprint' of each isolated component. Properly performed GC-MS analysis unequivocally identifies the compound, not just the drug class or chemical family. For the confirmatory analysis an extract from a new aliquot of urine is prepared using the same protocol as for the screening procedure, or using an appropriate alternative.

GC provides two elements of identification: RT and RRT. The GC retention time (RT) of a compound is the time elapsed between injection of the extract into the inlet and appearance of the compound at the outlet. It is reproducible under equal operating conditions. If an unknown has the same RT as the standard it may be the same substance or it may be a different one. If an internal standard is present in both the unknown extract and the standard extract, one can calculate the relative retention time (RRT) as: RT (compound)/RT (internal standard). The RRT is more reproducible than the RT. If an unknown has a RRT different from the RRT of the standard it is a different substance. The main determinant of RT and RRT is column polarity. Another means of characterizing a substance is retention index as originally described by Kovats [6] and later modified and improved by various authors [17]. This is useful because it allows comparison with the scientific literature.

Mass spectrometry is a very powerful technique, but many of its capabilities cannot be realistically applied on a large scale for routine work. The two most frequently employed ionization techniques are electron impact and chemical ionization. The electron impact mode is commonly used to obtain a full scan. Chemical ionization is particularly useful to confirm the molecular weight of a substance.

Most mass spectrometers can give an interpretable full scan spectrum with less than one nanogram of material. However even greater sensitivity can be achieved by monitoring only a few characteristic ions of the suspected compounds in the selective ion monitoring (SIM) mode. Data collected in the SIM mode may not be considered as sufficient proof of positive results by the most demanding chemists, nevertheless SIM data are commonly presented to document a positive finding and this is acceptable to many regulatory bodies and scientists. If the analysis is based on SIM data, the certainty is greatly enhanced if more than one characteristic substance is found in the sample, for example the parent drug and a metabolite. In addition if SIM data are used it is important to demonstrate equivalence between ion ratios for the sample and a standard. Furthermore one should demonstrate that the ion of interest dominates its region by monitoring the ions immediately preceding and following it. This shows that the pertinent ion is not derived from the preceding ion, and that the following ion is present in the proper ratio to the pertinent ion.

Unambiguous identification is accomplished by matching the RRT and spectra of the identified substances with those of authentic reference standards concurrently extracted from spiked urines (positive quality control) or certified positive cases. The reference spectra are contained in a mass spectral library. Such a library should be developed in each laboratory by analyzing derivatized compounds or their metabolites under comparable operating conditions. If reference standards of metabolites are not available, clinical studies may be performed by administration of the parent drug to man followed by timed urine collections, and the resulting urines used as positive quality control samples.

### Quality assurance

Quality assurance is 'planned and systematic actions necessary to provide adequate confidence that a product or service will satisfy given requirements for quality' [18,19]. Quality assurance encompasses quality control (QC) and quality assessment. The overall aim is to ensure that the analytical results are of sufficient accuracy for their intended application. The goal of quality assurance in drug testing is to minimize and document the probability of false positive and false negative results, as well as to document compliance with good laboratory practices (GLP) and the requirements of regulatory agencies. Quality is a team work ethic which materializes in various forms in all aspects of the work. Quality assurance may be implemented by a designated senior scientist and manager. Some laboratories refer to such individuals as QA officers.

In the context of large drug testing programmes, for example those operated by governments, sport authorities, and military establishments, quality is monitored by accreditation and proficiency testing programmes that establish requirements of competence and equivalency for the protection of the organization conducting the testing and of the individuals tested. Usually the requirements cover personnel needs and qualification and material resources (for example instruments) and analytical capabilities specific to a finite list of substances at specific concentrations. Compliance is documented by on-site inspection and written reports. Failure to comply results in immediate restriction of the work. The proficiency testing aspect of the programme utilizes biological samples prepared to contain known amounts of specific banned substances. The samples are sent to the laboratory in the blind (unknown to the laboratory) or open (known to the laboratory) mode. Proficiency testing programmes document the strengths and deficiencies of the participating laboratories, and provide objective data for between laboratory comparisons [20,21].

Critical review of QC data and optimization of QC design should be a constant and integral part of the work of a drug testing laboratory. Each laboratory maintains written Standard Operating Procedures (SOP) which describe each protocol in unique, detailed, and mandatory terms. Technicians are expected to adhere to the SOP. Laboratories should utilize internal QC biological samples either blind (to those doing the analyses) or open. Good quality control procedures monitor all possible sources of error and provide for rapid isolation of the problem. At least 10% of the samples in a batch are related to QC. Such procedures assess: clerical accuracy, sample preparation recovery, chromatographic performance, mass spectral sensitivity, overall assay detection threshold, and precision in quantitation. Examples of independent cross-checks are overlapping successive batches of QC samples or requiring verification by two persons. Record-keeping should include QC data and the description of measures taken to correct actual problems.

### Interpretation of test results

### *Pharmacokinetics*

In theory, complete knowledge of the pharmacokinetics of a drug enables a precise prediction of the concentration of drug in urine at various times after drug administration. In practice, however, many relevant variables cannot be known and the predictions become relatively gross estimates. Nevertheless, knowledge of the principles of pharmacokinetics enables the person responsible for interpreting the results of a drug test (the interpreter) to provide the most complete and accurate report and assessment.

The concentration of drug in plasma at various times after intravenous drug administration is determined by the dose and clearance (a pharmacokinetic variable that encompasses half-life and volume of distribution) [22]. The concentration in urine may also be estimated if clearance of drug and water are known. If the drug is orally administered the model must include bioavailability and the rate of absorption. Sophisticated pharmacokinetic models can also account for multiple and variable doses and dosage intervals [22,23]. Drugs with long half-lives and/or large volumes of distribution may be detectable in small amounts in urine for months. Examples of studies which provide useful pharmacokinetic data are: marijuana [24–26], cocaine [23,27], amphetamine [28]. Reviews [3,6,8] provide additional references.

Many of the relevant variables are rarely known, therefore the interpreter evaluates the pattern of excretion of drug and metabolites as determined by clinical studies. One must consider the sensitivity of the assays since it is a major determinant of detection times. Clearly a large dose will result in a positive test for a longer time than a small dose. Multiple closely spaced doses will result in drug accumulation, an increase in total body burden, and longer detection times. For example urine collected within the first few hours after cocaine administration contains both cocaine and benzoylecgonine, while urine collected a several hours later will not contain detectable amounts of cocaine [27]. The principal metabolite of tetrahydrocannabinol (11-nor-delta-9-tetrahydrocannabinol-9-carboxylic acid) is usually detectable for one or two days after single exposure, however multiple exposures may lead to a positive test for two or more weeks after cessation [25]. In addition passive exposure to marijuana smoke may result in sufficient absorption to result in detectable amounts of marijuana metabolites in urine [29].

Single doses of many anabolic steroids result in a positive test for between one and three days, while multiple doses result in positive tests for many days or weeks. Steroids that are formulated in oil and administered by injection may be detectable for several months.

### *Drug metabolism*

The majority of drugs are lipophilic and undergo oxidative metabolism resulting in more polar, ionizable metabolites which are either eliminated as such or after conjugation. Alterations in the rate and extent of drug metabolism influences the elimination half-life and clearance of drugs, and as a consequence, the concentrations of parent drug and of metabolites in blood and in urine.

About 90% of all oxidative metabolic reactions are catalysed by the cytochrome P450 enzyme system present in endoplasmic reticulum of liver cells, therefore this enzyme system plays a central role in the metabolism of lipophilic drugs such as stimulants, beta-blockers, steroids, opioids and other drugs.

Comprehensive knowledge of the metabolism of each banned substance is essential to the interpretation of drug testing results. For example the ingestion of some types of poppy seeds results in morphine in the urine [30]. Since morphine is a major metabolite of heroin, the detection of morphine is compatible with administration of heroin or the ingestion of poppy-seed products. One way to clarify this situation is to analyse the urine for 6-monoacetylmorphine, which is a metabolite of heroin and not a metabolite of morphine, and not found in poppy seeds [31].

### *Drug interactions*

The best studied drug interactions known to markedly influence drug or metabolite concentrations in body fluids are inductions and inhibitions of enzymatic reactions catalysed by cytochrome P450. Induction results in a decrease in half-life (increase in clearance) and more rapid elimination from the body. Furthermore induction often changes the pattern of metabolite elimination. Substances well known to induce the P450 system include drugs such as barbiturates, glutethimide, carbamazepine, ethanol and phenytoin (hydantoins) and environmental chemicals, such as polycyclic aromatic hydrocarbons (3-methylcholanthrene, benzo(a)pyrene) and polychlorinated aromatic hydrocarbons (polychlorinated biphenyls).

The inhibition of oxidative drug metabolic reactions by relatively unspecific inhibitors like cimetidine, chloramphenicol or sulfonamides will increase drug half-life and increase blood levels of the inhibited drug. This results in a decrease in the concentration of both parent drug and metabolites in urine, and therefore the ability to detect drug use. Furthermore the individual may experience enhanced drug effects and/or toxicity.

A number of clinically significant drug interactions resulting from concomitant administration of cimetidine with drugs such as caffeine, beta-blockers, barbiturates, morphine, and ethanol have been described [32]. The mechanism of inhibition is an interaction of cimetidine with the heme iron in cytochrome P450 [33]. Like cimetidine, the anabolic steroid stanozolol interacts with cytochrome P450 and is a potent inhibitor of cytochrome P450-catalysed reactions [34]. Depending of the particular isoenzymes of that are inhibited stanozolol could alter the elimination of other anabolic steroids.

Interactions involving drugs that influence the pH and water metabolism of the body alter the excretion rates of many banned drugs. The urinary excretion of many drugs is markedly influenced by urine pH [22,28,35,36]. The excretion of basic drugs (for example amphetamine) decreases as the pH of urine increases. Bicarbonate has been used to rapidly change the excretion rate of basic drugs and thereby reduce the likelihood of a positive test. Similarly the excretion of acidic drugs (for instance some diuretics) is retarded by low urinary pH. Other drugs influence the excretion of anionic substances. For example probenecid and related substances temporarily decreases the tubular excretion of anabolic steroids, penicillins, indomethacin and others. Some drug users attempt to avoid detections by adding adulterants directly to urine [15].

The state of hydration of an individual, by its effect on the water content of urine, markedly influences the concentration of drugs. Indeed, excessive intake of fluids is commonly used to dilute the urine and lower the concentration of drugs. Similarly diuretics have been used to rapidly dilute the urine. Some testing programmes routinely measure the specific gravity of urine to provide some insight into this problem. Measuring the urinary creatinine and reporting the concentration of drug per mg of creatinine is another technique of factoring for the effect of dilution.

Consideration of the pharmacokinetics, pharmacodynamics, metabolism, and drug interactions leads to the conclusion that it is very difficult to answer the question of whether or not an individual was under the influence of the drug at the time the sample was taken. Given sufficient analytical data and using tenable assumptions, the interpreter may be able to offer a reasonable opinion on this question.

### Documentation of results

The analytical report should be limited to statements of fact so as not to be confused with opinion. Therefore, where necessary, two distinct documents should be provided. The analytical results and other observations are normally documented in the analytical report, whereas matters of opinion should appear in a letter which accompanies that report.

### *Content of the analytical report*

The analytical report must provide sufficient information to enable the recipient to identify the individual(s) from whom the sample(s) described in the report originated. To avoid transcription errors all code numbers should be checked and double-checked by two persons. A complete description of the testing occasion is the best way to prevent such errors.

The report will contain chain-of-custody information including the date and time of arrival of the samples in the laboratory so that the analytical report may be linked to other chain-of-custody documentation. The integrity of the samples will be documented by including statements that the samples were sealed and, where appropriate, recording the seal numbers or other identifying features. The type(s) of assay performed must be stated either explicitly or, where an accepted protocol has been established, implicitly.

#### *Qualitative results*

Either the absence of the substances or the presence of the chemical entities identified in the individual samples must be clearly stated. Examples of appropriate statements might be:

> 'No substance banned by the (insert name of organization] was found in any of the samples.'
>
> 'The sample coded 2345A was found to contain [insert proper name] as described in the details attached. No substance banned by the [insert name of organization] was found in any of the other samples.'

The proper name should be the IUPAC name of the chemical entity identified or if the identified substance is a parent drug (as distinct from a metabolite of that drug) then the International Non-Proprietary Name (INN) might be used instead. Under some circumstances the use of the IUPAC name is cumbersome. In these cases, it may be convenient to use the INN.

It is customary for supporting data to be included, such as chromatographic data and GC-MS data obtained from the sample specified, and, in addition, data on corresponding reference standards (or reference urines obtained from suitable excretion studies). The reference data would normally be obtained after the specified sample has been analysed with suitable precautions to exclude the possibility of contamination. A sufficient description of the analytical methods used should be presented to enable the chromatographic and mass spectral data to be interpretable.

#### *Quantitative results*

Many drug testing programmes define a particular concentration of drug or metabolite as the dividing value for reporting a urine result positive or negative. This value, which is often referred to as the cut-off, represents an administrative decision on the part of the programme. The cut-off may be considerably greater than the detection limit (lowest concentration that can reliably be detected) of the assay. Cut-off concentrations may apply to the screening test and the confirmation test. For example the IA cut-off for cocaine in the US department of Defense program is 300 ng/ml and the corresponding cut-off for benzoylecgonine by GC/MS is 150 ng/ml [4]. This leads to the terminology 'analytical positive', which refers to a sample containing detectable amounts of drug, but which is reported or considered negative by the programme.

If the quantitative results are greater than the cut-off, at a minimum the sample is reported positive, or more informatively—substance detected. It is more informative to report that the substance was detected at a concentration greater than the cut-off. Clearly, the concentration must exceed the cut-off by more than the experimental variation. In all other cases, the mean of the measured values should be stated together with an estimate of the precision, for example relative standard deviation (coefficient of variation) or confidence interval for the measurements.

If quantitative results are being reported and the substances under investigation are present in concentration which are less than the cut-off value but greater than the detection limit, then the results should indicate that fact in the format of—substance detected, not greater than X (where X is the cut-off defined by the programme). In this case the sample is positive by analytical criteria, but negative by administrative criteria. It is informative to report the detection limit of the laboratory for that specific substance at that time. The substance should not be reported as being not present or even not detected.

*Supplemental information and opinion*

Avoid including in the analytical report irrelevant findings since they might be misinterpreted or confusing. Similarly, do not include in the analytical report the finding of endogenous or even ubiquitous substances except and unless the analytical protocol so demands, for example where concentration limits are imposed and/or where administration of a substance which is indistinguishable chemically from an endogenous substance is suspected (testosterone for instance).

Any additional information or statements of opinion may be expressed in a separate document, for example a covering letter. Examples of information which might be relevant include:

(1) Highlighting a declaration which has accompanied the samples such as a medical certificate, or drug declaration by the individual who has provided the sample where this is relevant and the laboratory data supports that statement.

(2) Where a chemical entity has been found and detailed in the analytical report, and the presence of this substance is considered to be a characteristic metabolite of a present drug subject to control, then an appropriate statement should be given with supporting literature references, for example '11-nor-delta-9-tetrahydrocannabinol-9-carboxylic acid is a metabolite of THC', or, 'the presence of 19-norandrosterone (3-alpha-hydroxy-5-alpha-estran-17-one) is considered to be evidence of the administration of the anabolic steroid nandrolone'.

No statement about the intent of the individual providing the sample should be made. The laboratory should restrict its report to the sample provided (other than as described above). It is generally unwise to make any unsolicited comments about the time when the last administrations might have occurred since there is unlikely to be any supporting information, for example dose taken, size of individual, metabolic characteristics of individual available to the laboratory at that time.

### Acknowledgements

We thank the following organizations and entities for supporting this work: Centre National de la Recherche Scientifique, Service Central D'Analyse (FR); the United States Olympic Committee, the National Collegiate Athletic Association (USA), the Norwegian Confederation of Sport (NOR), and the British Sports Council (GBR).

### References

1. Catlin, D. H., Kammerer, R. C., Hatton, C. K., Sekera, M. H. and Merdink, J. L., *Clinical Chemistry*, **33** (1987), 319.
2. Council of Europe. Anti-doping Convention (Council of Europe, Strasbourg, France, 1989), 1–19.
3. Hawks, R. L., in *Urine Testing for Drugs of Abuse*, Eds Hawks, R. L. and Chiang, N. C. (NIDA Res Monog 73. DHHS Pub. No. (ADM) 87-1481, Washington, D.C.: Supt. of Documents, U.S. Govt. Print. Office, 1986), 30.
4. Irving, J., *Clinical Chemistry*, **34** (1988), 637.
5. National Institute on Drug Abuse. *Mandatory Guidelines for Federal Workplace Drug Testing Programs* (Federal Register, 1988).
6. Substance-Abuse Testing Committee, *Clinical Chemistry*, **34**(3) (1988), 605.
7. Willette, R. E., in *Urine Testing for Drugs of Abuse*, Eds Hawks, R. L. and Chiang, N. C. (NIDA Res Monog 73. DHHS Pub. No. (ADM) 87–1481, Washington, D.C.: Supt. of Documents, U.S. Govt. Print. Office, 1986), 13.
8. Catlin, D. H. and Hatton, C. K., *Adv. Int. Med.*, **36** (1990), 381.
9. Masse, R., Ayotte, C. and Dugal, R., *Journal of Chromatography*, **489** (1989), 23.
10. Brooks, R. V., Jeremiah, G., Webb, W. A. and Wheeler, M., *Journal of Steroid Biochemistry*, **11** (1979), 913.
11. Donike, M., Barwald, K. R., Klostermann, K. *et al.* *Sport: Leistung und Gesundheit*, in Eds Heck, H., Hollmann, W. and Liesen, H. *et al.* (Koln, Kongressbd. Dtsch. Sportarztekongress, 1982), 293.
12. Chamberlain, R. T., *Clinical Chemistry*, **34** (1988), 633.
13. Dubin, C. L., Commission of inquiry into the use of drugs and banned practices intended to increase athletic performance. (Canadian Government Publishing Services, Ottawa, Canada).
14. Spiehler, V. R., O'Donnell, C. M. and Gokhale, D. V., *Clinical Chemistry*, **34** (1988), 1535.
15. Mikkelson, S. L. and Ash, K. O., *Clinical Chemistry*, **34** (1988), 2333.
16. Kovats, E., *Helvetica Chimica Acta*, **41** (1958), 1915.
17. Evans, M. B. and Haken, J. K., *Journal of Chromatography*, **472** (1989), 93.

18. ISO 9000, Quality management and quality assurance standards—Guidelines for selection and use. (International Standards Organization, Geneva, 1987).
19. Uldall, A., *Scandinavian Journal of Clincial Laboratory Investigation*, **47** (1987), 1.
20. Davis, K. H., Hawks, R. L. and Blanke, R. V., *Journal of the American Medical Association*, **260** (1988), 1749.
21. Hansen, H. J., Caudill, S. P. and Boone, J., *Journal of the American Medical Association*, **253** (1985), 2382.
22. Catlin, D. H., in *Essentials of Pharmacology*, Eds Bevan, J. A. and Thompson, J. H. (Philadelphia, 1983).
23. Chow, M. J., Ambre, J. J., Tsuen, I. R., Atkinson, A. J., Bowsher, D. J. and Fischman, M. W., *Clin. Pharm. Thera.*, **38** (1985), 318.
24. Bastiani, R. J., in *The Cannabinoids: Chemistry, Pharmacologic and Therapeutic Aspects*, Eds Agurell, S. *et al.* (Academic Press, New York, 1984), 263.
25. Law, B., Mason, P. A., Moffat, A. C., Gleadle, R. I. and King, L. J., *Journal of Pharm. Pharmacology*, **36** (1984), 289.
26. Wall, M. E., Sadler, B. M., Brine, D., Taylor, H. and Parez-Reyes, M., *Clin. Pharmacol. Ther.*, **34** (1983), 352.
27. Ambre, J., Tsuen, I. R., Nelson, J. and Belknap, S., *Journal of Analytical Toxicology*, **12** (1988), 301.
28. Beckett, A. H., Salmon, J. A. and Mitchard, M., *Journal of Pharm. Pharmacology*, **21** (1969), 251.
29. Cone, E. J. and Johnson, R. E., *Clin. Pharmacol. Ther.*, **40** (1986), 247.
30. Bjervert, K., Johnson, J., Nilsson, A. and Schuberth, J., *Journal of Pharm. Pharmacology*, **34** (1982), 798.
31. Mule, S. J. and Casella, G. A., *Clinical Chemistry*, **34** (1988), 1427.
32. Murray, M., *Drug Metabolism Review*, **18** (1987), 55.
33. Rendic, S., Kaifez, F. and Ruf, H. H., *Drug Metab. Disp.*, **11** (1983), 137.
34. Rendic, S. and Ruf, H. H., *Biochemical Pharmacology*, **37** (1988), 766.
35. Delbeke, F. T. and Debackere, M., *Arzneim-Forsch Drug Res.*, **36** (1986), 134.
36. Delbeke, F. T. and Debackere, M., *Arzneim-Forch Drug Res.*, **36** (1986), 1413.

